# Maternal Parental Self-Efficacy Following Child-Focused Birth Preparation Classes for Families Expecting a Second Child: A Pilot Exploratory Study

**DOI:** 10.3390/healthcare14010033

**Published:** 2025-12-23

**Authors:** Tomomi Tanigo, Sanae Marumoto, Masayuki Endo

**Affiliations:** 1 Faculty of Nursing, Osaka Dental University, 11-8 Hanazono-machi, Kuzuha, Hirakata-shi 573-1121, Osaka, Japan; 2 Division of Health Science, Graduate School of Medicine, Osaka University, 1-7, Yamadaoka, Suita-shi 565-0871, Osaka, Japan

**Keywords:** second child, multiparous mothers, childbirth preparation education, parental self-efficacy, pilot study

## Abstract

**Highlights:**

**What are the main findings?**

- The group receiving childbirth preparation education during pregnancy demonstrated elevated PSOC, RSES, and MAI scores.
- Participating mothers reported fostered confidence and ease in their parenting abilities.

**What are the implications of the main findings?**

- Childbirth preparation education improves maternal PSE postpartum.
- Preparation education facilitates a smoother transition to family life and fostered maternal confidence.

**Abstract:**

**Background/Objectives**: Mothers expecting a second child experience the parenting of multiple children for the first time, differing from first-time motherhood. This highlights the need for childbirth preparation education tailored to families expecting a second child. Parental self-efficacy influences maternal mental health, child development, and parent–child interactions. This non-randomized pilot exploratory study aimed to examine the association between childbirth preparation education for families expecting a second child and maternal parental self-efficacy at 1-month postpartum, focusing on a family-based, single-session program actively involving firstborn children. **Methods**: The intervention group (n = 18) received childbirth preparation education during pregnancy and completed questionnaires and semi-structured interviews at 1-month postpartum. The control group (n = 34) completed questionnaires only at 1-month postpartum. Questionnaires included the Parenting Sense of Competence Scale, Rosenberg Self-Esteem Scale, Maternal Attachment Inventory, Edinburgh Postnatal Depression Scale, and demographic information. Semi-structured interviews explored participants’ experiences and feelings after attending the childbirth preparation class. **Results:** Compared to the control group, the intervention group had higher Parenting Sense of Competence Scale scores; mothers in the intervention group reported smoother family-wide adaptation to life with a second child, greater confidence in child-rearing, recognition of the firstborn’s growth into an older sibling, and effective use of hands-on experiences from the class. **Conclusions**: Childbirth preparation education for families expecting a second child may be associated with higher maternal parental self-efficacy at 1-month postpartum. This association may reflect collective family preparation and adjustment supporting adaptation to life with a second child.

## 1. Introduction

The process of becoming a mother has been extensively studied [1]. Maternal identity evolves as mothers acquire new skills to respond to the emerging challenges of motherhood [2]. Therefore, the first-time motherhood experience differs from that of multiparous mothers, that is, mothers with two or more children. First-time and multiparous mothers adapt differently to childcare demands [3]; however, in both groups, maternal identity develops through skill acquisition and adaptation to changing family roles, according to Mercer’s theory of Becoming a Mother [2]. Although most studies have focused on first-time mothers, the transition to motherhood for mothers giving birth to a second child represents a distinct process, as they are required to adapt to caring for multiple children and reorganizing family routines [4]. Some studies have reported that parenting experiences do not differ significantly between multiparous and first-time mothers [5], indicating that these are qualitatively distinct experiences that cannot be directly compared. Additionally, multiparous mothers may experience higher stress than first-time mothers [6], with parity reportedly predicting maternal stress [7]. Mothers giving birth to a second child become parents of multiple children for the first time. These second-time mothers care for two children at different developmental stages, with the first child sometimes exhibiting regression or jealousy toward the second child [8]. Furthermore, when caring for two children simultaneously, mothers may feel guilt or confusion if they are unable to engage with both children equally or if the first child is restrained and the second child is left crying, a phenomenon known as “second child syndrome” [9,10]. However, most studies on the process of becoming a mother have focused on first-time mothers, with limited studies on mothers having a second child. Mothers expecting a second child experience anxiety about lifestyle changes, including the first child’s reaction to the birth of the second child and managing care for both children [11]. Hence, mothers expecting a second child experience a unique transition, balancing care for multiple children while adapting to changing family roles, which may generate stress and require specific skills and support. Therefore, childbirth preparation education specifically tailored to families expecting a second child, distinct from programs designed for first-time mothers, is needed.

Since 1980, sibling preparation classes (childbirth preparation education for older siblings) have been offered to support families with a second or subsequent child [12,13]; however, studies evaluating their effectiveness remain limited [14]. Reports on sibling preparation classes have indicated only small effects on children’s behavior [15,16]. One study revealed that children in the intervention group exhibited significantly reduced avoidance behaviors toward newborns by 4 months [17]. In Japan, qualitative evaluations of classes for families and older siblings expecting a second child have shown positive outcomes, including mothers’ increased acceptance of their first child’s regressive behaviors and recognition of the child’s growth [18,19].

Additionally, studies that provided childbirth preparation education to mothers and quantitatively assessed maternal psychological outcomes postpartum reported positive results, including the first child’s interest in the second child and mothers’ positive caregiving experiences [20]. It has been suggested that greater effects can be achieved when children participate in childbirth preparation education [12].

In pediatric nursing, preparation methods, including providing explanations appropriate to a child’s cognitive development regarding treatments or examinations and creating environments and opportunities that foster coping abilities in children and parents, are widely used [21,22]. Therefore, we hypothesized that providing childbirth preparation education to the entire family, including the first child, would help the first child prepare to accept the second child in the family.

Bandura [23] proposed the concept of self-efficacy in social learning theory. He argued that when an individual engages in a behavior, outcome expectations (believing a behavior will lead to the desired results) should be accompanied by efficacy expectations (believing that one can perform the behavior). Self-efficacy refers to an individual’s perception of their efficacy expectations [24].

PSE is a concept derived from Bandura’s self-efficacy, specifically applied to the parenting domain [24], and has been studied in psychology and nursing [25,26]. We focused on PSE as an outcome of support for mothers who had given birth to their second child. Bandura revealed that new skill acquisition is facilitated by self-efficacy. In stressful situations, the significance of self-efficacy in acquiring new skills is even greater [27]. PSE is associated with parental mental health, child development, and parent–child interactions, exerting positive effects [28] and highlighting its significance in parenting support.

PSE measurement is broadly categorized into two types, namely domain-specific measures (used to assess the efficacy of specific parenting behaviors, such as breastfeeding, bathing, or diaper changing) and domain-general measures (used to assess overall parenting efficacy). Mothers giving birth to a second child may have high domain-specific PSE for individual parenting tasks based on their experience with the first child; however, their domain-specific PSE for simultaneously caring for two children may be lower. Although scales exist for measuring domain-specific PSE for individual behaviors, no specific scale is available for multiparous mothers. Therefore, we measured the domain-general PSE.

Common tools for measuring domain-general PSE include the Parenting Sense of Competence Scale (PSOC) [29] and the Parenting Self-Agency Measure (PSAM) [26,30]. Comparisons between both tools indicate that the PSOC has been used at 1-month postpartum and comprises two subscales, Efficacy and Satisfaction, which have been applied separately in previous studies [26,31]. Although PSAM is another option, there is no Japanese version with established reliability and validity. Therefore, in this study, we used the PSOC to assess overall maternal PSE, noting that it does not specifically measure simultaneous care of two children and interpretation should be made with caution in such cases.

PSE has been theoretically defined as parents’ belief in the effectiveness of their parenting abilities and the perception of their ability to successfully raise their child [23,24,29]. Leveraging the two PSOC subscales, efficacy and satisfaction, we established an operational definition of PSE for this study. Specifically, PSE was defined as (1) behavioral competence, parents’ perception that their actions in childcare situations are effective, and (2) emotional satisfaction, parents’ sense of fulfillment derived from parenting.

Although previous studies have primarily focused on first-time mothers, little is known about the experiences of mothers expecting a second child, who must adapt to caring for multiple children and reorganizing family routines. Childbirth preparation education specifically tailored to families expecting a second child may support maternal adaptation and enhance parental self-efficacy (PSE), a key factor influencing maternal mental health, child development, and parent–child interactions. However, quantitative evidence on the effects of sibling-focused childbirth preparation programs, particularly those involving the first child, remains limited. This study aimed to examine the changes in maternal PSE resulting from childbirth preparation provided during pregnancy to families expecting a second child. Specifically, this exploratory pilot study aimed to examine whether participation in childbirth preparation education for families expecting a second child is associated with improved maternal PSE at 1-month postpartum. This study is novel in examining a family-based, single-session childbirth preparation education program for families expecting a second child, with a particular focus on firstborn children and evaluating its association with maternal parental self-efficacy.

## 2. Materials and Methods

### 2.1. Study Design

This non-randomized controlled trial involving two groups, namely intervention and control groups, was conducted as an exploratory pilot study.

#### 2.1.1. Study Design for Intervention Group

The intervention group comprised pregnant women, between 26 and 35 weeks of gestation, who were expecting a second child and visited an obstetric hospital in Osaka City between March and June 2024. The inclusion criteria were as follows: women with an uncomplicated pregnancy whose first child was of preschool age at the time of participation in the childbirth preparation class and who provided written informed consent to participate in the study.

The age criterion for the first child was established because the responses and behaviors of children of elementary school age or older differ from those of younger children, which may influence maternal psychological states. In this study, the ages of the first children ranged from 1 to 6 years. The exclusion criteria were the inability to read or write Japanese and plans to live separately from the first child postpartum. Mothers who did not live with their first child were excluded because they did not experience the first child’s reactions or the simultaneous care of the two children, which could affect maternal psychological outcomes.

#### 2.1.2. Study Design for Control Group

The control group comprised 34 mothers with two children who attended a 1-month postpartum checkup at the same obstetric hospitals in Osaka City where the intervention group was recruited, between March and September 2024. In contrast to the intervention group, which was recruited antenatally, the control group was recruited postpartum. The inclusion criteria for the control group were an uncomplicated pregnancy and postpartum course, written informed consent to participate in the study, and having a first child of preschool age at the time of participation. Mothers who could not read or write in Japanese were excluded from the study.

### 2.2. Measurement

At 1-month postpartum, the participants in the intervention group completed an anonymous web-based questionnaire. The survey included the Japanese versions of the PSOC [32], Rosenberg Self-Esteem Scale (RSES) [33], Edinburgh Postnatal Depression Scale (EPDS) [34], Maternal Attachment Inventory (MAI) [35], and basic demographic information (maternal age, employment status, child age and sex, family composition, and household income). The primary outcome of this study was maternal PSE, measured using the PSOC scale.

The Japanese version of the PSOC, originally developed by Gibaud-Wallston to measure domain-general PSE [36], is used in its 16-item form. In its revised version, revised by Johnston and Mash [29], one item was excluded owing to low factor loading. In this study, we developed a 12-item, two-factor Japanese PSOC version based on the version of Johnston and Mash [27] and verified its reliability and validity [32]. The PSOC is scored on a 6-point Likert scale from 1 (strongly disagree) to 6 (strongly agree), with total scores ranging from 12 to 72; higher scores indicate greater PSE.

The Japanese RSES version [33] is a 10-item scale rated on a 4-point Likert scale ranging from 1 (strongly disagree) to 4 (strongly agree), with higher scores indicating higher self-esteem. Similarly, the Japanese EPDS version is used for postpartum depression screening, with a cutoff score of 9 or higher indicating suspected postpartum depression [34]. The Japanese MAI version [35] comprises 26 items rated on a 4-point Likert scale from 1 (rarely) to 4 (almost always), with total scores ranging from 26 to 104 and higher scores indicating stronger maternal attachment.

The questionnaire was administered at 1-month postpartum because longitudinal studies up to 8 months postpartum have shown that PSOC scores reach their lowest point at that time [37]. In Japan, it is common for mothers to return to their parents’ home for support during the first month after childbirth, a cultural practice known as “satogaeri.” This period is particularly important for our study because it marks the time when mothers resume caring for both the newborn and their older child and begin to adjust family roles. Therefore, assessing maternal self-efficacy at this time allows us to capture the initial adaptation to caring for two children.

Additionally, the control group completed an anonymous web-based questionnaire at 1 month postpartum. The survey included the same measures used for the intervention group, with an additional question about whether the mothers wished to participate in childbirth preparation education.

Because this study spans the life event of the second child’s birth, baseline measurements before the intervention were not feasible.

### 2.3. Procedure

#### 2.3.1. Procedures for Intervention Group

##### Participant Recruitment and Study Procedures

Participants were recruited from an obstetric hospital in Osaka City (population 2.81 million) that handles approximately 700 deliveries annually and specializes in perinatal care. Researchers and hospital midwives distributed recruitment leaflets to pregnant women during routine prenatal checkups.

Childbirth preparation education was provided between 32 and 36 weeks of gestation. At 1-month postpartum, participants received a web-based questionnaire via email. Additionally, semi-structured interviews lasting approximately 30 min were conducted online or in a private room within the hospital to ensure privacy, focusing on the mothers’ feelings toward childcare.

The interview guide included the following questions: (1) “What events have occurred since attending the childbirth preparation class?” and (2) “How did you feel during these events?”.

##### Content of Childbirth Preparation Education

This childbirth preparation education program was a single-session, family-based intervention designed for families expecting a second child. The program focused on firstborn children and their mothers and aimed to enhance maternal parental self-efficacy during the transition to caring for two children. The program targeted pregnant women and their first child, with optional participation by fathers or other family members.

Classes were held between April and June 2024 on Saturdays from 11:00 a.m. to 12:00 p.m. Each session included up to three families and was conducted at the same obstetric hospital. To minimize variability in children’s developmental stage, the ages of the first children were considered when forming groups. Two midwives facilitated the sessions, with one providing explanations and the other supporting hands-on activities. This program was developed based on previous studies [18,19,20] and is novel in its hands-on, experiential activities involving firstborn children, which helped them prepare for a sibling and allowed mothers to observe and anticipate family dynamics, potentially enhancing maternal parental self-efficacy.

The contents of the childbirth preparation education program are presented in Table 1. It covers the course of pregnancy, labor, and delivery, as well as the physiology and care of newborns. Life-sized fetal and neonatal dolls were used to facilitate children’s understanding through hands-on experience, allowing them to touch and interact with the models in a safe environment. In addition, puppetry, storybooks, and illustrated materials about the birth of a baby were used to enhance comprehension, with explanations adjusted according to each child’s level of understanding, as children’s ages ranged widely in developmental stages from 1 to 6 years old.

An environment that encouraged children to speak freely was created, and participation was promoted using sticker sheets and handmade paper medals that the children could attach at designated points during the session. A booklet was provided for mothers and family members, covering typical reactions of the first child after the birth of a sibling, response strategies, common emotional changes experienced by mothers after the birth of the second child, and possible situations associated with caring for two children, along with suggested coping strategies. Because each session was a single 60 min program, we did not formally assess intervention fidelity, track fathers’ participation, or monitor booklet use or at-home practice. However, the program was delivered using standardized materials and a fixed timetable, ensuring that the general content and structure were largely consistent across sessions. The course was developed and implemented as part of routine clinical practice and is not currently available as a publicly accessible program.

#### 2.3.2. Procedures for Control Group

Participants in the control group were recruited from the same obstetric hospital as those in the intervention group. During the 1-month postpartum checkup, hospital midwives distributed leaflets containing the study explanation and a QR code linked to a web-based questionnaire. The participants completed an online survey.

### 2.4. Data Analysis

For the questionnaire survey results, descriptive statistics were used to analyze maternal age, parity, maternal education, maternal employment status, household income, and scores on each scale. Differences in background characteristics between the intervention and control groups were analyzed as follows: maternal age and the age of the first child were compared using an independent t-test, whereas maternal education, employment status, household income, and the first child’s sex were compared using the chi-square (χ^2^) test. Differences in PSOC, RSES, and MAI scores between the intervention and control groups were analyzed after testing for normality using the Shapiro–Wilk test. An independent t-test was used for normally distributed variables, and the Mann–Whitney U test was used for non-normally distributed variables. All tests were two-tailed, and data were reported to one decimal place. Additionally, *p*-values were reported to three decimal places, and the significance level was set at 5%. Statistical analyses were performed using IBM SPSS Statistics for Windows version 28.0 (IBM Corp., Armonk, NY, USA).

For the interview data, verbatim transcripts were created, and portions describing behavioral competence and emotional satisfaction were extracted based on the operational definitions. Subsequently, these portions were divided into the smallest meaningful units and coded. The coded data were compared and integrated to form subcategories, which were abstracted into broader categories using the assigned category names. Multiple researchers analyzed interview data to enhance interpretation validity.

### 2.5. Ethical Considerations

The study was conducted with the approval of the following ethics committees: the Ethics Committee for Intervention Studies at Osaka University Hospital (Approval No. 23370), the St. Barnabas Hospital Research Ethics Committee (Approval No. 00047), and the Ethics Committee of Osaka Dental University (Approval No. 111337).

In the intervention group, the researchers explained the study overview in writing and verbally. Informed consent was obtained by asking the participants to check the consent box on the application form at enrollment. For the control group, a study overview was provided in writing, and consent was given by checking the consent box at the beginning of the web-based questionnaire before responding.

## 3. Results

**Figure 1:**
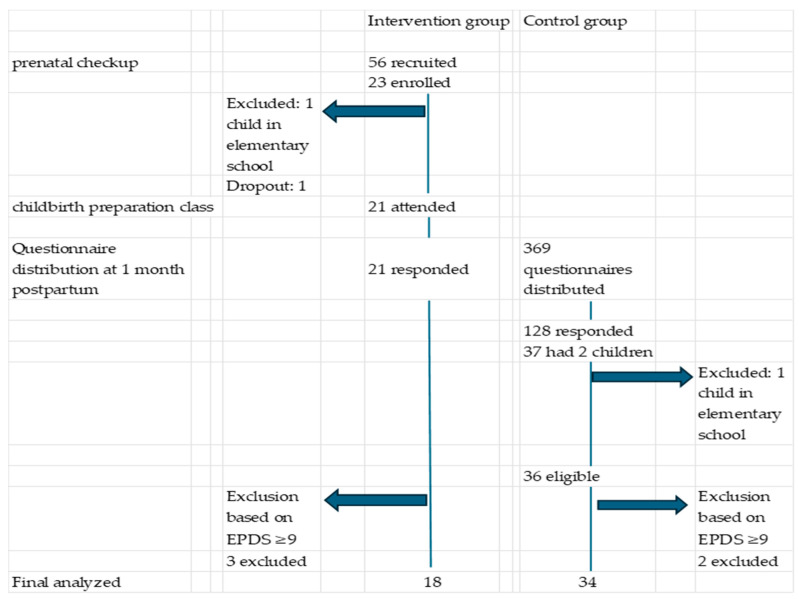
Flowchart of the study.

Study participant responses are shown in Figure 1. In the intervention group, 56 pregnant women were approached during prenatal health guidance sessions, and 23 agreed to participate in the study. The reasons for non-participation among the remaining 33 women were not collected. Among these, one participant was excluded because her first child was of elementary school age, and another was excluded owing to challenges regarding participation in the childbirth preparation class. Consequently, 21 participants in the intervention group completed the 1-month postpartum questionnaire and were included in the final analysis.

In the control group, questionnaires were distributed to 369 mothers at 1-month postpartum checkups, and 128 responses were obtained. Among these respondents, 36 mothers with one preschool-aged child met the inclusion criteria and were included in the control group. Because no previous studies have used PSOC scores to examine maternal PSE in this context, an a priori sample size calculation was not performed.

An EPDS score of nine or higher is used as the cutoff for suspected postpartum depression in Japan [34]. Participants in both groups with EPDS scores ≥ 9 were excluded from the analysis. The final sample included 18 and 34 mothers in the intervention and control groups, respectively.

The demographic characteristics of the 18 and 34 participants in the intervention and control groups, respectively, are presented in Table 2. No significant differences were observed between the groups regarding maternal age, first child age, maternal employment status, maternal education, household income, or first child sex.

**Table 2:** Background characteristics of study participants in the intervention and control groups.

Characteristic	Intervention Group (n = 18)	Control Group (n = 34)	*p*-Value
Maternal age (years), mean ± SD	33.5 (4.1)	33.7 (3.3)	0.824
Age of first child (years), mean ± SD	42 (14.7)	35 (15.7)	0.107
Maternal employment status			0.919
On maternity leave n(%)	13 (72.2)	25 (73.5)	
Not employed n(%)	5 (27.8)	9 (26.5)	
Maternal education			0.501
High school or below n(%)	2 (11.1)	2 (5.9)	
Junior college/University or higher n(%)	16 (88.9)	32 (94.1)	
Household income			0.690
<5 million yen n(%)	4 (22.2)	6 (17.6)	
≥5 million yen n(%)	14 (77.8)	28 (82.4)	
First child’s sex			0.178
Male n(%)	13 (72.2)	18 (52.9)	
Female n(%)	5 (27.8)	16 (47.1)	

Comparisons of the scale scores between the 18 and 34 participants in the intervention and control groups, respectively, are presented in Table 3.

**Table 3:** Comparison of maternal scores at 1-month postpartum in the intervention and control groups.

Measure	Intervention Group (n = 18)		Control Group (n = 34)		*p*-Value	Cohen’s d
	Mean	SD	Mean	SD		
PSOC	44.5	6.8	39.5	5.7	0.007 *^a^	0.816
RSES	38.7	4.3	35.0	7.4	0.029 *^b^	0.561
	Median	IQR	Median	IQR		Effect size (r)
MAI	96.0	88–104	91.0	75–107	0.005 *^c^	0.390

Notes: ^a^ Student’s *t*-test; ^b^ Welch’s *t*-test; ^c^ Mann–Whitney U test. * *p* < 0.05. PSOC, Parenting Sense of Competence Scale. RSES, Rosenberg Self-Esteem Scale. MAI, Maternal Attachment Inventory.

For PSOC scores, normality was assessed using the Shapiro–Wilk test. The intervention (*p* = 0.775) and control (*p* = 0.527) groups met the criteria for a normal distribution (*p* > 0.05). Homogeneity of variance was confirmed (F(1,50) = 0.656), and an independent Student’s t-test revealed significantly higher PSOC scores in the intervention group than in the control group (t(50) = −2.8, *p* = 0.007, Cohen’s d = 0.82).

For the RSES scores, the Shapiro–Wilk test indicated a normal distribution in both groups (intervention, *p* = 0.055; control, *p* = 0.237). Variance homogeneity was not confirmed(F(1,50) = 0.01), and Welch’s t-test showed significantly higher RSES scores in the intervention group than in the control group (t (50) = −2.2, *p* = 0.029, Cohen’s d = 0.56).

The Shapiro–Wilk test results for MAI scores were *p* = 0.056 and *p* = 0.023 for the intervention and control groups, respectively, indicating non-normal distribution in the control group. Accordingly, the Mann–Whitney U test was conducted, and significantly higher MAI scores were observed in the intervention group than in the control group (*p* = 0.005, effect size r = 0.39).

When asked whether they would have liked to participate in childbirth preparation education for their first child and family members during pregnancy, 20 mothers in the control group responded affirmatively, whereas 14 did not. No significant differences in PSOC (39.75 ± 5.71 vs. 39.21 ± 5.87, t = 0.27, *p* = 0.79), RSES (35.3 ± 7.75 vs. 34.64 ± 7.06, t = 0.25, *p* = 0.80), or MAI (90.5 [70.5–110.5] vs. 91.0 [75.0–107.0], *p* = 0.820, Mann–Whitney U test) were observed between these subgroups.

The qualitative interviews were conducted alongside the quantitative assessment. Although formal data saturation was not fully assessed, consistent themes were observed across participants, providing insights into maternal experiences. PSE in the intervention group at 1 month postpartum is presented in Table 4. We extracted 272 codes, 21 subcategories, and 4 categories from the verbatim transcripts.

At 1-month postpartum, mothers in the intervention group reported ***adapting to a new family life with the baby as a whole family, gradually realizing that the older child has grown beyond expectations and truly becomes an older sibling, utilizing the hands-on experiences from the class in the whole family’s new life with the baby, and feeling confident and at ease in child-rearing as the new life has been going well.***

One mother stated, “I felt I could face child-rearing with a sense of security because I had already discussed with my partner that he would mainly play outdoors with the older child, and that we would switch when the older child wanted to play with me while I cared for the baby.” Thus, she experienced being *able to adjust roles within the family and face child-rearing with a sense of security.* Another mother said, “When the baby cried, the older child tried to soothe the baby or immediately came to get me. I thought she might be too absorbed in her own things to pay attention to the baby, but she acted more maturely than I imagined. I really feel her growing up.” She experienced *feeling growth in the older child’s attitude and behavior.* Another mother said, “When we participated in the class, I saw that the older child was a bit rough when touching the baby doll and didn’t quite understand how to handle it yet. It helped me imagine how she would interact with the real baby after birth, which I think was really helpful.” This mother experienced *participation in the class allowed preparation for handling the older child, balancing roles, and family coordination*. Additionally, another mother said, “Before the baby was born, I anticipated how much I would be able to do postpartum, how much attention the baby would need, and what care the older child still required. I planned what to ask my husband and mother for help with. Therefore, now that we have two children, it feels like everything is running smoothly.” This mother experienced *feeling that parenting of both children is going well through family preparation and adjustments*.

## 4. Discussion

### 4.1. Effects of Childbirth Preparation Education on Maternal PSE: Quantitative and Exploratory Findings

As an exploratory pilot study with a small sample size, the findings should be interpreted cautiously. When childbirth preparation education was provided primarily to the first child during the mother’s second pregnancy, and maternal PSOC scores were compared at 1-month postpartum, the intervention group scores were significantly higher than those of the control group (Cohen’s d = 0.82, indicating a large effect). Although this strong association suggests potential benefits of the intervention, the non-randomized design of the current study prevents definitive conclusions about causality. Nevertheless, the observed effect size provides useful preliminary data for designing and calculating the sample size of a future large-scale randomized controlled trial (RCT), highlighting the value of this study as a proof-of-concept. Furthermore, while previous studies have examined childbirth preparation classes and firstborn behaviors, the effects on maternal PSE in second-time mothers have not been explored. This study therefore provides preliminary evidence that a family-based, single-session childbirth preparation class may enhance maternal PSE shortly after birth, representing a novel contribution to the literature.

Moreover, although no previous studies have directly examined maternal PSE in second-time mothers, some studies have reported that firstborn children’s avoidant behaviors toward the newborn decreases after childbirth preparation classes. While our study did not measure firstborn avoidance, the observed increase in maternal PSE may be related to these behavioral changes, suggesting that family-based childbirth preparation supports both maternal confidence and sibling adjustment.

Mothers expecting a second child reportedly feel uncertain about how to explain the birth of a sibling to their first child [11]. The interview results revealed that mothers believed that their first children’s hands-on experiences, including seeing and touching dolls, facilitated their understanding. This suggests that conducting childbirth preparation education with experiential activities tailored to the child’s developmental stage helped the first child understand and prepare for the birth of a sibling, which may have contributed to the enhancement of maternal PSE.

Similarly, mothers reported that observing their child’s reactions in class and their interactions with the neonatal doll helped them visualize life after birth and prepare their family for the new arrival. The booklet provided to mothers at the end of the class included guidance on interacting with the first child, situations likely to arise after the second child’s birth and how to handle them, and changes in maternal emotions.

Bandura noted that PSE is strengthened by successful experiences in similar situations [38]. Previous studies have reported that by holding a neonatal doll and observing the first child’s reactions, mothers can prepare for interactions with the first child and recognize their emotions, facilitating a smoother adaptation after the second child’s birth [39]. In the present study, mothers participated in the class with their first child and reviewed the booklet after, allowing them to observe their first child’s reactions, which may have helped mothers anticipate postpartum experiences and may have contributed to the observed increase in PSE.

At 1-month postpartum, mothers in the intervention group had significantly higher RSES (Cohen’s d = 0.56) and MAI (r = 0.39) scores, suggesting that the education may have had a moderate effect. The first child reportedly undergoes a transition to siblinghood (TTS) from before the birth of a younger sibling until approximately 1 year after birth, showing psychological and behavioral changes, such as anxiety, depressive symptoms, and aggressive behaviors. However, the degree varies depending on environmental and individual factors, such as temperament [40]. Additionally, previous studies have reported that the behaviors of the first child when the mother holds a neonatal doll are not necessarily predictive of the behaviors the first child will exhibit after the actual birth of the newborn [41]. In this study, it is possible that participation in the childbirth preparation class helped the first child prepare in becoming an older sibling, which may have mitigated some of the psychological and behavioral changes typically observed during TTS. Furthermore, as described above, mothers could visualize life after birth, which may have promoted emotional composure, enhanced self-esteem, and strengthened their attachment to their second child. Given the intervention’s single session, the durability of the observed effects beyond 1 month postpartum remains unknown.

### 4.2. Qualitative Insights into Maternal PSE and Family Adaptation

In this study, the PSOC scores of the intervention group were 5 points higher than those of the control group. Here, we discuss the PSE of mothers in the intervention group. Mothers in the intervention group reported gradually realizing that their older child had grown beyond expectations and had truly assumed the role of an older sibling. Previous studies [10] have shown that even mothers who did not receive childbirth preparation education experienced similar feelings, such as being pleased with the unexpected growth of the first child or being happy that the first child showed more interest in and engaged with the younger sibling than anticipated.

However, in this study, mothers also reported that the hands-on experiences in the class were used by the entire family to navigate life with the new baby, suggesting that these experiences contributed to the first child’s growth and facilitated family-level preparation and coordination. Consequently, mothers perceived the process of adapting to a new family life with their baby as a shared family experience. Using the class as a trigger, the family collectively prepared for the arrival of the second child, even before birth, which facilitated adaptation to a new life. This aligns with Mercer’s Becoming a mother theory [3], which states that maternal identity develops through skill acquisition and adaptation to changing family roles.

Additionally, mothers reported feeling confident and experiencing a sense of ease in child-rearing as the new life was progressing smoothly, which may indicate that the family’s successful adaptation contributed to their maternal reassurance and confidence. We believe that the difference in PSE between the intervention and control groups may be related to the intervention group’s family collectively prepared for the second child’s birth before delivery, which may have facilitated adaptation to the new family life.

### 4.3. Clinical Implications and Future Research of Childbirth Preparation Education for Families Expecting a Second Child

The findings of this study suggest the potential benefits of childbirth preparation education for families expecting a second child; however, the participants were limited to late-pregnancy mothers living in a single city in Japan who attended small, resource-rich classes with access to perinatal services. Therefore, caution is warranted when generalizing to other regions or contexts.

This study revealed that providing childbirth preparation education to families expecting a second child during pregnancy may improve maternal PSE at 1-month postpartum. From the second pregnancy through the early postpartum period, the arrival of a new family member represents a joyful event for the mother, first child, and father, while also constituting a major environmental change and a potentially highly stressful situation.

In such high-stress situations, PSE may be particularly important for acquiring new parenting skills, such as simultaneously caring for two children [25]. It is assumed that the participants in this study were highly influenced by PSE for mothers expecting a second child; an increase in PSE may facilitate the acquisition of new parenting skills, which is meaningful for promoting successful parenting.

Moreover, PSE affects maternal mental health, child development, and parent–child interactions [26], suggesting that enhancing maternal PSE after the birth of a second child may positively affect skill acquisition and the well-being of parents and children. This study is also novel in incorporating hands-on, experiential activities for firstborn children, allowing mothers to observe family interactions and potentially enhancing maternal PSE. Therefore, childbirth preparation education for families expecting a second child may improve maternal PSE at 1-month postpartum and contribute to better parenting, suggesting the potential value of incorporating such programs into standard care.

However, participatory childbirth preparation classes involving first children require small group sizes and significant personnel resources, which may limit their implementation in many facilities. Disseminating these findings and exploring more efficient, feasible operational methods may be important for future studies and clinical practice.

### 4.4. Limitations

This study has several limitations. First, as an exploratory pilot study, the sample size was small, and no prior PSOC scores were available to inform sample-size calculation, limiting the statistical power of the findings. Because the birth of a second child is a major life event, maternal PSE may naturally decrease postpartum. In future studies, both intervention and control groups should ideally be recruited during pregnancy, and pre-intervention measurements should be collected to compare changes in PSE between groups and to examine causal relationships more robustly. Second, selection bias may have influenced the results, because mothers in the intervention group were self-selected and possibly more motivated, which itself could have contributed to higher maternal PSE. In addition, because PSOC data for the control group were only collected postpartum, it remains unclear whether the observed differences were owing to the intervention or owing to pre-existing group differences. However, no significant differences were observed between the intervention and control groups in basic maternal characteristics, such as age and educational level, suggesting that the observed effects are attributable to the intervention. Third, uncontrolled confounding factors—such as family support (including “satogaeri”), optional attendance by fathers or other family members, and differences in recruitment timing between groups—may have affected maternal adjustment and the observed outcomes. Data on fathers’ or other family members’ participation and support were not systematically recorded in this study; future research should explicitly collect such information to examine its potential influence on maternal outcomes. Fourth, the study was conducted in a single hospital in Japan, and cultural and institutional factors may limit the generalizability of the findings to other regions or healthcare settings. Fifth, the PSOC scale used does not specifically measure maternal self-efficacy for simultaneous care of two children. Future research should consider developing a scale tailored to this context.

## 5. Conclusions

Providing prenatal childbirth preparation education to families expecting a second child may enhance maternal PSE at 1-month postpartum compared with a control group. Mothers in the intervention group reported that education facilitated family-level preparation and adjustment for the new baby, enabling a smoother transition to family life and fostering maternal confidence and psychological ease in parenting. These results suggest that childbirth preparation education specifically tailored for families expecting a second child may be beneficial for improving maternal PSE postpartum.

## Figures and Tables

**Table 1:** Contents of Childbirth Preparation Education.

Time	Content	Materials Used	Teaching Points
Before—10 min after start	Free picture book reading	15 picture books about the baby’s birth	- Allow the first child to become used to the venue and staff, and be ready to listen to explanations
3 min	Orientation	Challenge sheet	- Explain today’s program content - Give 1 sticker per story; reward given if 3 stickers are collected - Encourage children’s participation
10 min	Pregnancy process Hands-on holding experience	Picture cards, Fetal dolls (7 dolls from zygote to fetal stage matching gestational weeks)	- Show events and fetal size corresponding to mother’s gestational week - Check children’s concentration - Arrange all dolls to show the growth process - Explain the umbilical cord function and that the mother was once a fetus
15 min	Labor process	Picture cards, Puppet play	- Explain vaginal opening - Explain going to the hospital when contractions start (class location) - Explain contractions as a pushing force for baby’s birth - Explain the role of doctors and midwives - Explain cesarean section - Explain “we were also born this way” - Explain postpartum hospitalization and rest - Explain that parental love remains unchanged after birth
15 min	Newborn care experience	Koken baby, Disposable diapers	- Explain the anterior fontanelle and undeveloped neck - Compare own body with the baby’s to feel the size difference - Experience holding the baby to feel its weight - Diaper changing and dressing according to the child’s developmental level
2 min	Summary	Medal, Booklet distribution	- Give a medal when three stickers are collected to enhance achievement - Distribute booklet to parents
5 min		Free time/reflection	- Allow free use of materials and books - Provide individual guidance, ask for impressions, and answer questions
Total: 60 min			

**Table 4:** Parental self-efficacy in the intervention group at 1-month postpartum.

Category	Subcategory
Adapting to a new family life with the baby as a whole family	Able to adjust roles within the family and face child-rearing with a sense of security
	Able to organize family routines and environment in a way that works for our household
	Understanding and responding to the older child’s feelings together as a family
	Working together as a family with a shared approach to child-rearing
Gradually realizing that the older child has grown beyond expectations and truly becomes an older sibling.	The older child participates in baby care more than expected
	Feeling growth in the older child’s attitude and behavior
	Feeling that the older child has become aware of being an older sibling
	Feeling reassured that the older child shows interest in the baby and interacts on their own initiative
	Feeling that the older child interacts with the baby at an appropriate distance
	Feeling that the older child is balancing their own feelings and interest in the baby
	The older child’s regressive behaviors, jealousy, or confusion are milder than expected and can be managed by the family
Utilizing the hands-on experiences from the class in the whole family’s new life with the baby	Hands-on experience improved the older child’s understanding of how to interact with the baby
	Through experience, the older child developed an interest in and accepted the baby
	Participation in the class allowed preparation for handling the older child, balancing roles, and family coordination
	Hands-on experience helped the mother visualize how the older child behaves and how family life will be with the new baby
	The class experience provided an opportunity for the family to talk about welcoming the baby
Feeling confident and at ease in child-rearing as the new life has been going well	Experience with the older child helps the mother feel more confident and relaxed
	Feeling joy in seeing the baby’s cuteness and observing the interaction between the older child and the baby
	Balancing ideal child-rearing with actual life leads to satisfaction with current parenting
	Feeling that parenting of both children is going well through family preparation and adjustments
	Developing a sense of being a four-person family with the new baby

## Data Availability

The data presented in this study will be made available upon request by the corresponding author owing to privacy and ethical restrictions.

## Abbreviations

The following abbreviations are used in this manuscript:

PSE	parental self-efficacy
PSOC	Parenting Sense of Competence Scale
RSES	Rosenberg Self-Esteem Scale
EPDS	Edinburgh Postnatal Depression Scale
MAI	Maternal Attachment Inventory
TTS	transition to siblinghood

## References

[1] Rubin R. (1984). Maternal Identity and the Maternal Experience.

[2] Mercer R.T. (2004). Becoming a mother versus maternal role attainment. J. Nurs. Sch..

[3] Gameiro S., Moura-Ramos M., Canavarro M.C. (2009). Maternal adjustment to the birth of a child: Primiparity versus multiparity. J. Reprod. Infant. Psychol..

[4] Nelson A.M. (2003). Transition to motherhood. J. Obs. Gynecol. Neonatal Nurs..

[5] Krieg D.B. (2007). Does motherhood get easier the second-time around? Examining parenting stress and marital quality among mothers having their first or second child. Parenting.

[6] Mortazavi F., Chaman R., Mousavi S.A., Khosravi A., Ajami M.E. (2013). Maternal psychological state during the transition to motherhood: A longitudinal study. Asia Pac. Psychiatry.

[7] Skari H., Skreden M., Malt U.F., Dalholt M., Ostensen A.B., Egeland T., Emblem R. (2002). Comparative levels of psychological distress, stress symptoms, depression and anxiety after childbirth—A prospective population-based study of mothers and fathers. BJOG.

[8] Volling B.L. (2012). Family transitions following the birth of a sibling: An empirical review of changes in the firstborn’s adjustment. Psychol. Bull..

[9] Young P.C., Boyle K., Colletti R.B. (1983). Maternal reaction to the birth of a second child: Another side of sibling rivalry. Child. Psychiatry Hum. Dev..

[10] Tomomi T., Michiko M., Minako S. (2015). Mothers’ experiences and thoughts on raising two children in the first three months following the birth of a second child. Jpn. J. Matern. Health.

[11] Beyers-Carlson E., Schoenebeck S., Volling B.L. (2022). Mother of One to Mother of Two: A Textual Analysis of Second-Time Mothers’ Posts on the BabyCenter LLC Website. Front. Psychol..

[12] Parma S. (1979). A family centered event? Preparing the child for sharing in the experience of childbirth. J. Nurse Midwifery.

[13] Johnsen N.M., Gaspard M.E. (1985). Theoretical foundations of a prepared sibling class. J. Obs. Gynecol. Neonatal Nurs..

[14] Kramer L., Ramsburg D. (2002). Advice given to parents on welcoming a second child: A critical review. Fam. Relat..

[15] Wilford B., Andrews C. (1986). Sibling preparation classes for preschool children. Matern. Child. Nurs. J..

[16] Fortier J.C., Carson V.B., Will S., Shubkagel B.L. (1991). Adjustment to a newborn. Sibling preparation makes a difference. J. Obs. Gynecol. Neonatal Nurs..

[17] Beyers-Carlson E.E.A., Volling B.L. (2017). Efficacy of Sibling Preparation Classes. J. Obs. Gynecol. Neonatal Nurs..

[18] Kataoka Y., Sutou H., Nagamori K., Horiuchi S. (2008). Impacts of a sexuality education class on children, pregnant mothers and their family: Changes in mothers’ feelings, concerns and family response. J. Jpn. Acad. Midwif.

[19] Nakamura A., Kataoka Y., Horiuchi S., Tsuchiya M., Tanaka S., Yajima C. (2006). Implementing and evaluating a new sibling preparation class for children and parents. J. Jpn. Acad. Midwif.

[20] Akemi I. (2016). Development and evaluation of a preparatory education program for mothers having their second child. J. Jpn. Acad. Midwif.

[21] Lööf G., Lönnqvist P.A. (2022). Role of information and preparation for improvement of pediatric perioperative care. Paediatr. Anaesth..

[22] Copanitsanou P., Valkeapää K. (2014). Effects of education of paediatric patients undergoing elective surgical procedures on their anxiety—A systematic review. J. Clin. Nurs..

[23] Bandura A. (1977). Self-efficacy: Toward a unifying theory of behavioral change. Psychol. Rev..

[24] Bandura A. (1997). Self-Efficacy; the Exercise of Control.

[25] Montigny F., Lacharité C. (2005). Perceived parental efficacy: Concept analysis. J. Adv. Nurs..

[26] Jones T.L., Prinz R.J. (2005). Potential roles of parental self-efficacy in parent and child adjustment: A review. Clin. Psychol. Rev..

[27] Bandura A. (1982). Self-efficacy mechanism in human agency. Am. Psychol..

[28] Albanese A.M., Russo G.R., Geller P.A. (2019). The role of parental self-efficacy in parent and child well-being: A systematic review of associated outcomes. Child. Care Health Dev..

[29] Johnston C., Mash E.J. (1989). A measure of parenting satisfaction and efficacy. J. Clin. Child. Psychol..

[30] Dumka L.E., Stoerzinger H.D., Jackson K.M., Roosa M.W. (1996). Examination of the cross-cultural and cross-language equivalence of the parenting self-agency measure. Fam. Relat..

[31] Wittkowski A., Garrett C., Calam R., Weisberg D. (2017). Self-Report Measures of Parental Self-Efficacy: A Systematic Review of the Current Literature. J. Child. Fam. Stud..

[32] Tanigo T., Endo M., Ohashi K. (2024). Development of a Japanese Version of the Parenting Sense of Competence Scale, and Examining the Structure of Japanese Mothers’ Parenting Self-Efficacy. Children.

[33] Yamamoto M., Matui Y., Yamanari Y. (1982). The structure of perceived aspect of self. Jpn. J. Educ. Psychol..

[34] Okano T., Murata M., Fusako M., Tamaki R., Nomura J., Miyaoka H., Kitamura T. (1996). Validation and reliability of a Japanese version of the EPDS. Arch. Psychiatirc Diagn. Clin. Eval..

[35] Nakajima T. (2001). Reliability and Validity of the maternal attachment Inventory. J. Jpn. Acad. Nurs. Sci..

[36] Gibaud-Wallston J., Wandersman L.P. (1978). Development and Utility of the Parenting Sence of Competence Scale.

[37] Mercer R.T., Ferketich S.L. (1995). Experienced and inexperienced mothers’ maternal competence during infancy. Res. Nurs. Health.

[38] Bandura A. (1995). Exercise of Personal and Collective Efficacy in Changing Societies. Self-Efficacy in Changing Societies.

[39] Chapman J.K., Hart S.L. (2017). The transition from mother-of-one to mother-of-two: Mothers’ perceptions of themselves and their relationships with their firstborn children. Infant. Ment. Health J..

[40] Zhang Q., Wu W., Sheng L., Xi X., Zhou Y., Wen Y., Liu Q. (2023). Emotional and Behavioral Changes in Preschool Firstborn Children During Transition to Siblinghood: A Mixed Methods Study. Psychol. Res. Behav. Manag..

[41] Volling B.L., Bae Y., Rosenberg L., Beyers-Carlson E.E.A., Tolman R.M., Swain J.E. (2022). Firstborn Children’s Reactions to Mother-Doll Interaction Do Not Predict Their Jealousy of a Newborn Sibling: A Longitudinal Pilot Study. J. Perinat. Educ..

